# Plasma metabolome analysis for predicting antiviral treatment efficacy in chronic hepatitis B: diagnostic biomarkers and therapeutic insights

**DOI:** 10.3389/fimmu.2024.1414476

**Published:** 2024-07-12

**Authors:** Deying Chen, Yingfeng Lu, Jiangshan Lian, Jiong Yu, Liang Li, Lanjuan Li

**Affiliations:** ^1^ State Key Laboratory for Diagnosis and Treatment of Infectious Diseases, National Clinical Research Center for Infectious Diseases, Collaborative Innovation Center for Diagnosis and Treatment of Infectious Diseases, The First Affiliated Hospital, College of Medicine, Zhejiang University, Hangzhou, China; ^2^ The Metabolomics Innovation Centre and Department of Chemistry, University of Alberta, Edmonton, AB, Canada

**Keywords:** metabolomics, chronic hepatitis B, antiviral therapy, predictive biomarkers, immune response

## Abstract

The early and accurate identification of predictive biomarkers for antiviral treatment efficacy remains a significant clinical challenge, particularly in the management of chronic hepatitis B (CHB). This study aimed to assess whether the plasma metabolome could reliably predict the success of antiviral therapy in CHB patients. We conducted a retrospective analysis on 56 treatment-naive CHB patients at the First Affiliated Hospital of Zhejiang University from December 2013 to March 2016. Patients who underwent a 48-week treatment regimen of entecavir (ETV) and interferon-alpha (IFN-α) were randomly assigned to either a discovery cohort (n=29) or a validation cohort (n=27). Based on the outcome of the treatment, patients were classified as HBeAg seroconversion group (High responders, Hrp) or the non-remission group (Low responder, Lrp). Our methodology involved an untargeted analysis of the amine/phenol and carboxylic acid submetabolomes in the CHB patients under treatment, utilizing chemical isotope labeling (CIL) techniques with liquid chromatography-mass spectrometry (LC-MS). Several metabolites were identified as having significant diagnostic potential for distinguishing Hrp from Lrp, with areas under the receiver operating characteristic curve (AUC) exceeding those typical clinical indicators. Notably, four metabolites, namely 2-methyl-3-ketovaleric acid, 2-ketohexanoic acid, 6-oxo-1,4,5,6-tetrahydronicotinic acid, and α-ketoisovaleric acid, demonstrated exceptionally high sensitivity and specificity in both cohorts, nearing 100%. In contrast, the clinical indicators, including HBcAb, log(HBsAg), and HBeAb, demonstrated lower and inconsistent sensitivity and specificity between the discovery and validation cohorts. Using HBcAb as a marker, the sensitivity was 87.5% with 76.9% specificity in the discovery cohort; however, the sensitivity dropped to 46.7% with 91.7% specificity in the validation cohort. Using log(HBsAg), the sensitivity was 84.6% with 69.2% specificity in the discovery cohort, compared to 85.7% sensitivity and 83.3% specificity in the validation cohort. For HBeAb, the separation of Hrp and Lrp had a sensitivity of 87.5% with 69.2% specificity in the discovery cohort, while the validation cohort showed 86.7% sensitivity and 91.7% specificity.

## 1 Introduction

Chronic hepatitis B (CHB) presents a significant global public health challenge, affecting an estimated 257 million people worldwide. It is a leading cause of liver cirrhosis and hepatocellular carcinoma (HCC) (1). Hepatitis B e-antigen (HBeAg) seroconversion is recognized as a crucial marker for achieving HBV suppression and delaying disease progression in CHB patients (2). The current treatment regimen for CHB includes nucleoside analogs (NAs) and interferon-alpha (IFN-α). NAs, such as lamivudine, adefovir, telbivudine, entecavir, and tenofovir, work by suppressing HBV replication through the inhibition of viral DNA polymerase. However, these treatments rarely lead to HBsAg seroconversion (3). IFN-α therapy, on the other hand, has been shown to induce HBsAg seroconversion in a minority of CHB patients (4, 5). Combination therapy involving IFN has been found more effective in treating CHB patients when compared to nucleoside analog monotherapy, although seroconversion is achieved in only a limited subset of patients (6–8). To this end, the pursuit of optimized, individualized treatment and management strategies becomes crucial for enabling the majority of CHB patients to achieve a functional cure.

In this retrospective study, we analyzed the outcomes of patients treated with a combination of interferon (IFN) and nucleos(t)ide analogs (NA) at our hospital between January 2014 and December 2016. The patients were divided into two groups based on their 48-week clinical outcomes: those who achieved HBeAg seroconversion (High Responders, Hrp) and those who did not (Low Responders, Lrp). The aim of our study was to investigate whether observing changes in metabolic patterns before or during antiviral therapy could provide insights into the impact of treatments on metabolic processes. We hypothesized that identifying patients who are likely to respond to specific antiviral or immunomodulatory treatments using efficacy prediction biomarkers, either before or during the course of therapy, could improve rates of HBeAg and hepatitis B surface antigen (HBsAg) seroconversion, as well as HBsAg seroclearance. We believe that the development of personalized treatment strategies based on these biomarkers is a promising approach to enhance treatment efficacy and optimize healthcare resource utilization in the management of chronic hepatitis B. A targeted treatment strategy, which accurately selects patients who are more likely to respond well to particular treatment regimens, is expected to increase cure rates compared to a generalized treatment model. By implementing personalized treatment plans based on predictive biomarkers, healthcare providers can allocate resources more effectively and improve overall patient outcomes.

In this work, we utilized a chemical isotope labeling (CIL) liquid chromatography-mass spectrometry (LC-MS) metabolomics platform to characterize the amine/phenol and carboxylic acid submetabolomes. In CIL LC-MS, various labeling chemistries were employed to profile different submetabolomes, resulting in a more comprehensive coverage of the metabolome with accurate relative quantification of individual metabolites (9). Our analysis encompassed nearly 3000 metabolites through the examination of these two submetabolomes. Several metabolites were identified to have substantial diagnostic value in differentiating between Hrp and Lrp, demonstrating significantly higher areas under the receiver operating characteristic curve (AUC) than those observed with clinical markers. A set of metabolites were found to be associated with HBeAg seroconversion in individuals with chronic hepatitis B. We observed changes in key metabolites that could potentially support anti-HBV innate immunity or anti-inflammatory effects, providing new perspectives for treating and preventing inflammatory diseases. Patients with favorable treatment responses exhibited activated immune systems and gradually controlled inflammation levels over time. Given that the hepatic inflammatory reaction in CHB patients can lead to sustained damage and fibrosis of liver tissue, it is critical to understand the molecular mechanisms and metabolic regulation of the hepatic inflammatory response during treatment. Gaining these insights will be crucial for developing new and optimized treatment strategies and interventions that can help more patients achieve a functional cure.

## 2 Materials and methods

### 2.1 Patient cohort and study design

The study included 56 patients diagnosed with chronic hepatitis B (CHB) at the First Affiliated Hospital of Zhejiang University in Hangzhou, China, between December 2013 and March 2016. Patients were randomly assigned to a discovery cohort (n=29) and a validation cohort (n=27). All patients showed clinical, biochemical, and virological evidence of chronic HBV infection, evidenced by persistent plasma HBsAg positivity for over six months and anti-HBcAg antibody positivity. These patients had not received any prior antiviral treatment and were clinically diagnosed as CHB patients according to the ‘Guidelines for the Prevention, Care, and Treatment of Persons with Chronic Hepatitis B Infection’ (10). Exclusion criteria included patients with cirrhosis, concurrent liver cancer, metabolic diseases such as diabetes and hyperthyroidism, co-infection with hepatitis C, other forms of viral hepatitis, HIV infection, neurological or psychiatric abnormalities, and allergies to interferon or entecavir. The research protocol was approved by the hospital’s human ethics committee, and informed consent was obtained from all participants.

The study involved administering immunotherapy and interferon drugs to all participants for a duration of 48 weeks. The treatment plan included the use of the first-line antiviral drug entecavir (ETV) as a nucleotide analog and the selection of interferon alpha (IFN-α) as an immunomodulator. The drug regimen consisted of subcutaneous injections of PegIFNα at a dose of 180 µg once a week, and entecavir at doses of 0.5 mg/d. Follow-up assessments were conducted at baseline, 24 weeks, and 48 weeks after the initiation of treatment. The main observational indicators included changes in symptoms and signs, hepatitis B markers, HBV DNA levels, liver function, kidney function, blood routine, and the monitoring and recording of relevant adverse reactions.

Fasting blood samples were collected at designated time points and stored for subsequent analysis. Virological response was defined as a reduction in HBV DNA levels to below the detection limit of 20 IU/mL. HBeAg seroclearance was characterized by a reduction in HBeAg levels to below the detection threshold, with or without HBeAg seroconversion (defined as ≤ 1 S/CO), accompanied by the emergence of HBeAb (hepatitis B e-antibody). Based on the status of HBeAg seroconversion following treatment, patients were classified into two groups: ‘High Responder’ (Hrp) for those who achieved seroconversion, and ‘Low Responder’ (Lrp) for those who did not.

### 2.2 Biochemical tests

Clinical and laboratory data were retrospectively collected from outpatient medical records in accordance with standard biochemical tests and guidelines. Parameters for hepatic and renal function, including albumin (Alb), alanine aminotransferase (ALT), aspartate aminotransferase (AST), alkaline phosphatase (ALP), gamma-glutamyl transferase (GGT), total bilirubin (TB), direct bilirubin (DBIL), total bile acids (TBA), blood urea nitrogen (BUN), and creatinine, were measured using an automated biochemical analyzer. For immunological assessments, markers of hepatitis B virus (HBV) infection, such as hepatitis B e-antigen (HBeAg), hepatitis B e-antibody (HBeAb), hepatitis B surface antigen (HBsAg), hepatitis B surface antibody (HBsAb), and hepatitis B core antibody (HBcAb), were quantified using an automated chemiluminescence analyzer. Quantification of HBV DNA was performed with a quantitative real-time PCR analyzer. HBV DNA levels were specifically measured using the Roche COBAS TaqMan HBV Test, Version 2.0, which has a detection limit of 20 IU/mL. HBeAg levels were determined using an enzyme immunoassay capable of detection of as low as 1 S/CO. HBsAg quantification was conducted with the Elecsys HBsAg II assay (Roche Diagnostics, Germany), which has a sensitivity of down to 0.05 IU/mL. Complete blood counts, including white blood cell (WBC) count, platelet (PLT) count, and hemoglobin (HGB) levels, were analyzed with automated hematology analyzers.

### 2.3 Metabolomics analysis

Plasma samples stored at -80 °C were thawed at room temperature for one hour. For each sample, two aliquots of 30 µL were prepared: one for dansylation and the other for DMPA (p-dimethylaminophenacyl) labeling. To precipitate proteins, a threefold volume of ice-cold methanol was added to each aliquot. The mixtures were then centrifuged at 18,000 g for 30 minutes at room temperature. The clear supernatant was carefully transferred to a new 0.5-mL plastic vial and subsequently dried using a SpeedVac vacuum concentrator in preparation for chemical labeling.

Dansylation and DMPA labeling were carried out in accordance with established protocols to profile the amine/phenol (11) and carboxylic acid submetabolomes (12), respectively. Normalization of sample amounts was achieved by measuring the total UV absorbance of the dansyl-labeled metabolites on a Waters ACQUITY UPLC system (13). Concentrations of labeled metabolites were quantified for each sample using a calibration curve based on amino acid standards.

For the LC-MS analysis, an equal molar quantity of each individual ^12^C-dansyl or DMPA labeled sample was mixed with a ^13^C-dansyl or DMPA labeled reference pool. The mixtures were analyzed using reversed-phase LC-QTOF-MS on a Bruker Impact II instrument under previously established conditions (14). Metabolite peak pairs with ^12^C/^13^C labels were identified in the mass spectra, with peak intensity ratios providing a measure of concentration differences between individual samples and the reference pool. As the same reference pool was employed for all samples, the peak ratio values reflected relative concentration variations among the samples. To ensure quality control, a QC sample comprising equal moles of ^12^C- and ^13^C-labeled pooled samples was run after every 20 sample analyses.

### 2.4 Data processing and analysis

In the CIL LC-MS approach, ^12^C/^13^C-dansyl or DMPA labeled metabolites were detected as peak pairs. The extraction of these metabolite peak pairs, alignment across samples, and retrieval of missing values were performed using the IsoMS Pro software (15). Metabolite identification was done in a three-tiered metabolite identification approach as previously described (16). Volcano plot analysis was conducted as an effective and easy-to-understand graphical method that summarizes both fold-change and significance. It is a scatter plot that displays the negative log10-transformed p-values from the t-test against the log2 fold-change. Additionally, multivariate principal component analysis (PCA) and partial least squares (PLS) modeling were carried out using the software SIMCA 12+ and MetaboAnalyst for comprehensive data interpretation.

## 3 Results

### 3.1 Basic clinical characteristics of patients

This study’s discovery cohort, showed in **Table 1A**, provides a detailed overview of the demographic and clinical characteristics of the participants, tracked at the onset of the study at baseline (T0), at 24 weeks (T24), and at 48 weeks (T48). The discovery cohort was divided based on the clinical outcomes observed at 48 weeks into two distinct groups. The Hrp group, with an average age of 31.3 ± 7.0 years, was comprised of 11 men and 5 women, while the Lrp group had an average age of 29.3 ± 7.5 years, including 11 men and 2 women.

**Table 1:** Demographic and clinical characteristics of the study population in (A) discovery cohort and (B) in validation cohort, tracked at the onset of the study (0 weeks), at 12 weeks (T12), and at 48 weeks (T48).

Table 1(A)	Discovery set, Hrp (n=16)			Discovery set, Lrp (n=13)		
Age, mean ± SD	31.3 ± 7.0			29.3 ± 7.5		
Age, range	20–46			19–43		
Males,n(%)	11(69)			11(85)		
	Week 0	Week 24	Week 48	Week 0	Week 24	Week 48
HBsAb(MIU/mL)	3.7 ± 4.7	12.9 ± 36.5	15.9 ± 55.9	0.4 ± 0.6	0.5 ± 0.5	1.8 ± 5.3
HBcAb (S/CO)	13.4 ± 2	11.1 ± 2.5*	11 ± 3.1*	11.2 ± 1.1	10.6 ± 1.5	11.3 ± 1.7
HBeAg(PEIU/mL)	173.1 ± 162.9	11.8 ± 40.1*	0.2 ± 0.2*	315.9 ± 213.4	95.9 ± 105.2*	641.3 ± 2049.2
HBeAb(IU/mL)	27.9 ± 23.8	2.8 ± 6.8*	0.5 ± 0.6*	54.3 ± 22.8	26 ± 20.1*	15.7 ± 16.5*
log(HBsAg) IU/mL	3.6 ± 1.2	2.4 ± 1.5*	2.6 ± 1.1*	4.4 ± 0.5	3.9 ± 0.5*	3.8 ± 0.4*
log(HBV) IU/mL	6.9 ± 1.4	Not detect*	Not detect*	7.9 ± 0.9	4.4 ± 0.9*	4.5 ± 1.4*
Alb (g/L)	47.4 ± 4.2	47.4 ± 3.9	46.3 ± 3.7	45.8 ± 3	47.8 ± 2.3	47.1 ± 2.5
Glob (g/L)	28.5 ± 4.4	28.5 ± 4.1	28.2 ± 3.7	26.4 ± 1.8	28.1 ± 2.5	28.1 ± 3
ALT(U/L)	203.4 ± 257.9	35.2 ± 26.2*	25.3 ± 12.8*	132.5 ± 107.2	35.2 ± 16*	35.8 ± 17.1*
AST(U/L)	118.6 ± 144.5	26.2 ± 11.4*	22.1 ± 6.5*	67.1 ± 35.7	26.8 ± 10.5*	28.1 ± 11.2*
ALP(U/L)	83.3 ± 23.2	67.2 ± 15.4*	64.7 ± 14.7*	76.4 ± 23.7	72.8 ± 17.5	63.8 ± 13.7
GGT(U/L)	61.3 ± 47.1	26.4 ± 22.5*	25.3 ± 16.5*	49.8 ± 35.1	32.2 ± 17.6	27.2 ± 17.4
TBIL(µmol/L)	15.1 ± 5	9.4 ± 3.7*	9.2 ± 3.3*	12.8 ± 3.1	10 ± 3.2*	9.1 ± 2.9*
TBA(µmol/L)	14.4 ± 29	10.4 ± 14.3	15.1 ± 33.5	6.5 ± 4.7	7 ± 9.1	9.2 ± 11.2
BUN(mmol/L)	4.7 ± 0.9	4.2 ± 0.9	4.4 ± 0.9	3.9 ± 1	3.9 ± 0.8	4.1 ± 0.9
CR(µmol/L)	70.5 ± 14.2	70.6 ± 11.8	68.1 ± 14.4	68.4 ± 11.6	65.2 ± 11	64 ± 11.2
WBC(10 E9/L)	5.7 ± 1.7	5.3 ± 2.1	4.8 ± 1.5	5.7 ± 0.9	4.6 ± 1.8	4.4 ± 1.4
PLT(10 E9/L)	187.3 ± 61.4	160.7 ± 55.6	145.3 ± 51.2	192.8 ± 30.1	144.5 ± 27.7	167 ± 47.5
HGB(g/L)	145 ± 15.8	139.9 ± 17.6	133.9 ± 17.6	150 ± 16.4	140.4 ± 22.4	133.8 ± 21.5

Table 1(B)	Validation set, Hrp (n=15)			Validation set, Lrp (n=12)		
Age, mean ± SD	28.3 ± 4.2			30.1 ± 5.1		
Age, range	22–35			22–36		
Males,n(%)	9(60)			10(83)		
	T0	T24	T48	T0	T24	T48
HBsAb(MIU/mL)	0.8 ± 1.4	1.0 ± 1.4	1.0 ± 1.1	0.4 ± 0.5	0.4 ± 0.4	1.4 ± 3.7
HBcAb (S/CO)	12.9 ± 1.5	11.9 ± 1.0*	11.6 ± 1.1*	11.4 ± 1.7	11.3 ± 1.1	11.2 ± 1.9
HBeAg(PEIU/mL)	80.8 ± 124.4	0.8 ± 0.9*	0.2 ± 0.3*	446.6 ± 370.6	127 ± 124.7*	148.6 ± 122.6*
HBeAb(IU/mL)	13.6 ± 18.2	0.6 ± 0.6*	0.3 ± 0.6*	64.9 ± 23.9	26 ± 20.7*	23.7 ± 16.7*
log(HBsAg) IU/mL	4.0 ± 0.6	3.0 ± 0.7*	3.1 ± 0.9*	4.7 ± 0.4	3.9 ± 0.5*	3.8 ± 0.4*
log(HBV) IU/mL	7.3 ± 1.0	Not detect*	Not detect*	7.6 ± 1.9	4.8 ± 1.4*	5.1 ± 1.7*
Alb (g/L)	46.6 ± 3.7	48 ± 2.8	45 ± 2.6	47.2 ± 2.3	48.1 ± 2.6	45.5 ± 3.2
Glob (g/L)	27.7 ± 3.1	27.6 ± 3.1	27.4 ± 4.9	28.2 ± 3.9	27.8 ± 4.0	28.6 ± 5.5
ALT(U/L)	177.5 ± 123.3	40.9 ± 35.4*	30.5 ± 26.2*	150.9 ± 107.2	45.2 ± 28.7*	40.3 ± 49.2*
AST(U/L)	94.3 ± 54.7	29.9 ± 18.4*	23.1 ± 8*	69.2 ± 42.2	30.0 ± 13.0*	24.2 ± 16.1*
ALP(U/L)	81.6 ± 22.8	67.1 ± 13.1*	59.4 ± 10.6*	83.9 ± 32.6	69.9 ± 19.3	64.5 ± 22.3
GGT(U/L)	52.3 ± 23.4	36.1 ± 39.7	25.4 ± 23.9*	50.8 ± 38.9	45.6 ± 47.9	32.6 ± 37.0
TBIL(µmol/L)	18.5 ± 6.4	11.7 ± 2.9*	11.5 ± 4.8*	14.8 ± 4.4	11.5 ± 4.7	11.3 ± 3.6*
TBA(µmol/L)	11.7 ± 11.5	6.3 ± 4.2	5.9 ± 5.0	4.4 ± 3.8	4.4 ± 3.0	5.4 ± 3.7
BUN(mmol/L)	4.3 ± 1.3	3.9 ± 1.3	4.0 ± 0.8	4.6 ± 1.3	4.5 ± 1.4	4.4 ± 1.0
CR(µmol/L)	63.7 ± 16.5	66.4 ± 14.7	66.1 ± 16.2	79.1 ± 12.1	77.8 ± 16.7	78.5 ± 20.2
WBC(10 E9/L)	5.8 ± 1.4	5.0 ± 1.1	4.9 ± 1.2	6.3 ± 2.2	5.0 ± 1.3	4.9 ± 1.4
PLT(10 E9/L)	198.1 ± 66.4	145.5 ± 47.2	152.8 ± 40.2	189.8 ± 45	138.1 ± 52.5	156.4 ± 49.5
HGB(g/L)	148.7 ± 12.9	140.5 ± 17.3	130.8 ± 18.8	159.8 ± 10.2	149.8 ± 9.9	146.2 ± 14.5

This table includes patient data on the following clinical indicators: HBsAb, Hepatitis B Surface Antibody; HBcAb, Hepatitis B Core Antibody; HBeAg, Hepatitis B e-Antigen; HBeAb, Hepatitis B e-Antibody; HBsAg, Hepatitis B Surface Antigen; HBV DNA, Hepatitis B Virus DNA; Alb, Albumin; Glob, Globulin; ALT, Alanine Aminotransferase; AST, Aspartate Aminotransferase; ALP, Alkaline Phosphatase; GGT, Gamma-Glutamyl Transferase; TBIL, Total Bilirubin; TBA, Total Bile Acid; BUN, Blood Urea Nitrogen; CR, Creatinine; WBC, White Blood Cell count; PLT, Platelet count; HGB, Hemoglobin.

*P < 0.05 compared with control (T0).

Clinical observations revealed significant decreases in HBeAb, HBsAg, HBV DNA, ALT, and AST levels at both 24 weeks (T24) and 48 weeks (T48) of antiviral treatment in the Hrp group when compared to baseline (T0). It was found that HBV DNA levels became undetectable by week 24 of treatment in the Hrp group, and this undetectable status persisted until the end of the treatment period at week 48. The undetectable HBV DNA levels serve as a direct measure of effective viral replication suppression, a key indicator of successful antiviral treatment. Conversely, the Lrp group showed a significant 3-log reduction but did not reach undetectable levels, indicating less effective viral suppression. The initial higher levels of HBeAg observed in the Lrp group suggest a correlation between elevated baseline HBeAg levels and poorer treatment responses. Similarly, the lower HBeAg levels in the Hrp group before starting therapy could indicate a lower viral disease burden, which may have potentially contributed to their more favorable treatment outcomes. These findings suggest a relationship between baseline HBeAg levels and treatment efficacy, with lower pre-treatment HBeAg levels associated with better responses to therapy. In the later discussion, we will separately compare the clinical indicators and metabolic markers to predict differences in responses to antiviral therapy, with the goal of identifying sensitive and specific markers to predict treatment efficacy.

At the 24-week, both groups experienced significant reductions in HBeAg levels, with the Hrp group showing a more pronounced decrease (93% reduction in Hrp versus 70% in Lrp). This demonstrates a stronger response to therapy in the Hrp group in terms of both viral suppression and antigen level reduction. Additionally, the continuous decline in ALT levels throughout the treatment in both groups signifies a substantial reduction in liver inflammation among chronic hepatitis B (CHB) patients. However, the Lrp group saw an increase in HBeAg levels during the later stages of treatment, with no further reduction in HBV DNA levels, indicating a plateau in the treatment’s effectiveness. Despite these challenges, the initial 24 weeks of antiviral immune therapy yielded positive therapeutic effects for both groups, highlighting the potential for early intervention and tailored treatment strategies in managing CHB. The demographic and clinical characteristics of patients of the validation cohort (**Table 1B**) were, in general, quite similar to those described for the discovery cohort.

### 3.2 Metabolome data analysis

We applied CIL LC-MS for the targeted screening of the amine/phenol and carboxylic acid submetabolomes in patients with chronic hepatitis B (CHB) during treatment, considering that metabolites containing amine, phenol, or acid functional group(s) are involved in many metabolic pathways. This technique was preferred for its ability to mitigate matrix effects and ion suppression, which often complicate conventional LC-MS analyses (17). The incorporation of differential isotope labeling enhances the measurement of metabolic changes with improved accuracy and precision. We included peak pairs that appeared in over 80% of the samples within each group for statistical analysis. Combining the two cohorts’ metabolome data, we identified 1,398 peak pairs or metabolites within the amine/phenol submetabolome and 1,506 peak pairs or metabolites within the carboxylic acid submetabolome. Detailed results are presented in **Supplementary Table S1**. A tier 1 and tier 2 approach facilitated the positive identification of 658 metabolites. Additionally, matches were found for 658, 1,008, and 310 peak pairs within the zero-, one-, and two-reaction MyCompoundID libraries, respectively. Thus, out of the 2,904 peak pairs detected, 2,634 (90.7%) were either positively identified or matched. This extensive coverage enabled us to monitor metabolic changes throughout the treatment process, offering the potential to discover biomarkers that could be used to refine personalized treatment strategies and enhance the likelihood of achieving a functional cure for a broader segment of patients with chronic hepatitis B (CHB).

### 3.3 Multivariate comparison of metabolic changes during antivirus-therapy

**Figure 1:**
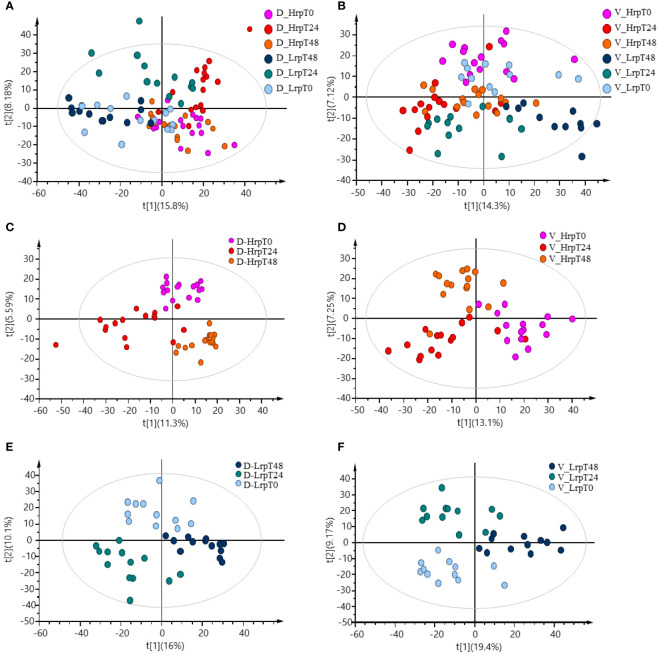
PLS-DA plots demonstrating the data-driven separation and clustering of two patient cohorts, discovery **(A)** and validation **(B)**, at three time intervals, excluding QC samples. Time-point specific plots for the Hrp group in the discovery **(C)** and validation **(D)** sets, and for the Lrp group in the discovery **(E)** and validation **(F)** sets.

Principal component analysis (PCA) offers an unsupervised approach to investigate multivariate differences among sample groups at specific time points. **Supplementary Figure S1** displays PCA scores plots for the discovery cohort (A) and the validation cohort (B), demonstrating the multivariate distribution of sample groups at distinct time intervals: baseline (T0), after 24 weeks (T24), and after 48 weeks (T48), along with quality control (QC) samples. The tight clustering of the QC samples underscores the methodological consistency throughout the analyses. After excluding QC samples, the partial least squares discriminant analysis (PLS-DA) plots were generated and presented in **Figures 1A, B**, illustrating the metabolomic data-driven separation and clustering of two distinct patient cohorts: Hrp and Lrp observed at three different time intervals, baseline (T0), 24 weeks (T24), and 48 weeks (T48). These two plots effectively demonstrate how, over the course of treatment, the Hrp groups form tight clusters within a specific region, whereas the Lrp groups are more widely dispersed in a neighboring area, with some overlap observed between the two groups. The Hrp groups are denoted by a bright color scheme, while the Lrp groups are represented by a darker palette. This color differentiation provides a clear visual distinction that mirrors the metabolic variances observed between the CHB patient groups. Panels C and D of **Figure 1** show the plots for the Hrp group across baseline (T0), 24 weeks (T24), and 48 weeks (T48) time points in the discovery and validation sets, respectively. This allows visualization of clustering and separation of the Hrp group samples at different stages of treatment in the two independent cohorts. Similarly, Panels E and F of **Figure 1** illustrate the plots for the Lrp group across the same time intervals in the discovery and validation sets, respectively. These four panels trace the metabolic trajectories of the Hrp and Lrp groups in both cohorts throughout the antiviral therapy and provide a graphical representation of the metabolic evolution in response to the treatment.

### 3.4 Cross-sectional and longitudinal analysis

We will divide the subsequent analysis into two parts. Part one (Cross-sectional study) focuses on searching for predictive biomarkers of antiviral treatment efficacy by analyzing the differences in the metabolome of patients with varying levels of immune response before receiving antiviral treatment (T0), selecting potential biomarkers related to treatment efficacy, and drawing ROC curves to evaluate the accuracy and reliability of the model. Part two (Longitudinal study) focuses on the analysis of the dynamic changes in the metabolome during antiviral treatment by studying the dynamic changes in the metabolome of patients with different levels of immune response during antiviral treatment, understanding the relationship between changes in the metabolome and treatment efficacy, and drawing a new perspective for treatment monitoring.

#### 3.4.1 Cross-sectional study: searching for predictive biomarkers of antiviral treatment efficacy

**Figure 2:**
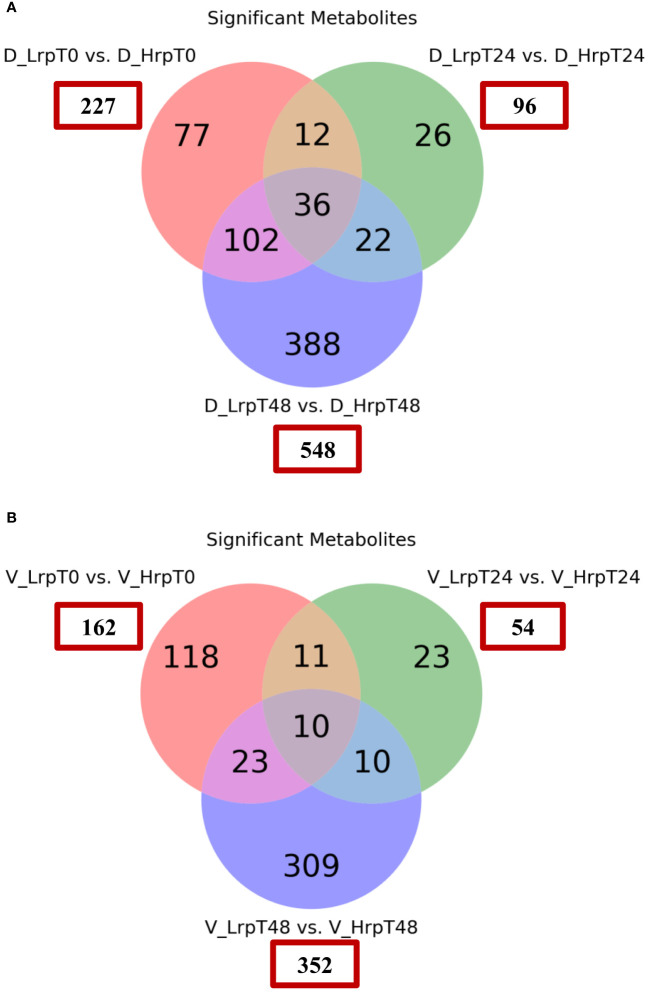
Venn diagrams showing the count of significantly differentially expressed metabolites between the Hrp and Lrp groups at baseline (T0), 24 weeks (T24), and 48 weeks (T48) for both the discovery **(A)** and validation **(B)** cohorts. The metabolites were considered significant if they met the following criteria: a fold change of ≥ 1.5 or ≤ 0.67 and a false discovery rate controlled at 5% using a q-value ≤ 0.05.

In this part, binary comparisons between the two treatment groups were conducted using a fold-change threshold of either ≥1.5 or ≤0.67, controlling the false discovery rate at 5% (q-value ≤0.05). In the discovery cohort, this analysis identified significant differences in the number of metabolites at baseline (0 weeks), mid-treatment (24 weeks), and end--treatment (48 weeks), with counts of 227, 96, and 548 significant metabolites, respectively, in the Hrp group compared to the Lrp group. Similarly, in the validation cohort, the analysis uncovered significant differences in the number of metabolites at the same time points, with 162, 54, and 352 significant metabolites, respectively, in the Hrp group compared to the Lrp group. These results are depicted in **Figure 2** using a Venn diagram. While there were differences in the metabolome level between the two groups before the start of treatment in both cohorts (227 vs. 162), by week 24, the difference in the number of differential metabolites between the two groups was more than halved in both cohorts (96 vs. 54). However, a significant increase was observed at the subsequent 48 weeks in both cohorts (548 vs. 352). This aligns with clinical treatments. Both groups showed improvement in clinical indicators at 24 weeks, but the improvement was more pronounced in the Hrp group. A possible explanation for this is that at 24 weeks of antiviral therapy, both groups exhibited a decline in HBV DNA, HBeAg, and HBsAg levels, mitigating the metabolomic differences between the groups. However, in the later stages of treatment, the Lrp group experienced a rebound.

**Figure 3:**
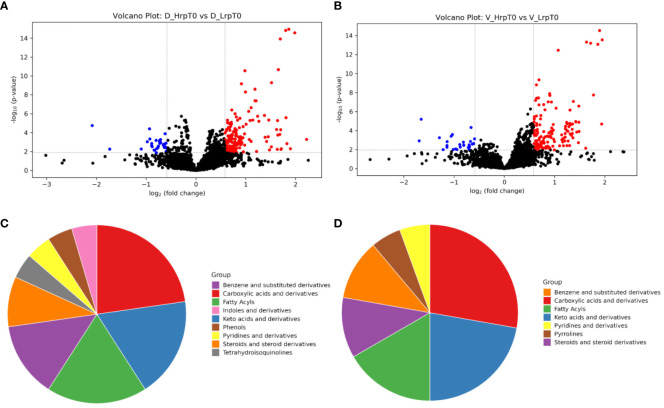
The volcano plots and pie charts visualize the differences in plasma metabolic profiles between patients with high immune response (Hrp) and low immune response (Lrp) at baseline (T0). Panels **(A, B)** present the volcano plots for the discovery and validation cohorts, respectively. These plots display the -log10(P-value) on the y-axis and the log2(fold change) on the x-axis between the two groups. Panels **(C, D)** show the percentage distribution of metabolite classes that are significantly different in the plasma of Hrp patients compared to Lrp patients for the discovery and validation cohorts, respectively.

**Figure 4:**
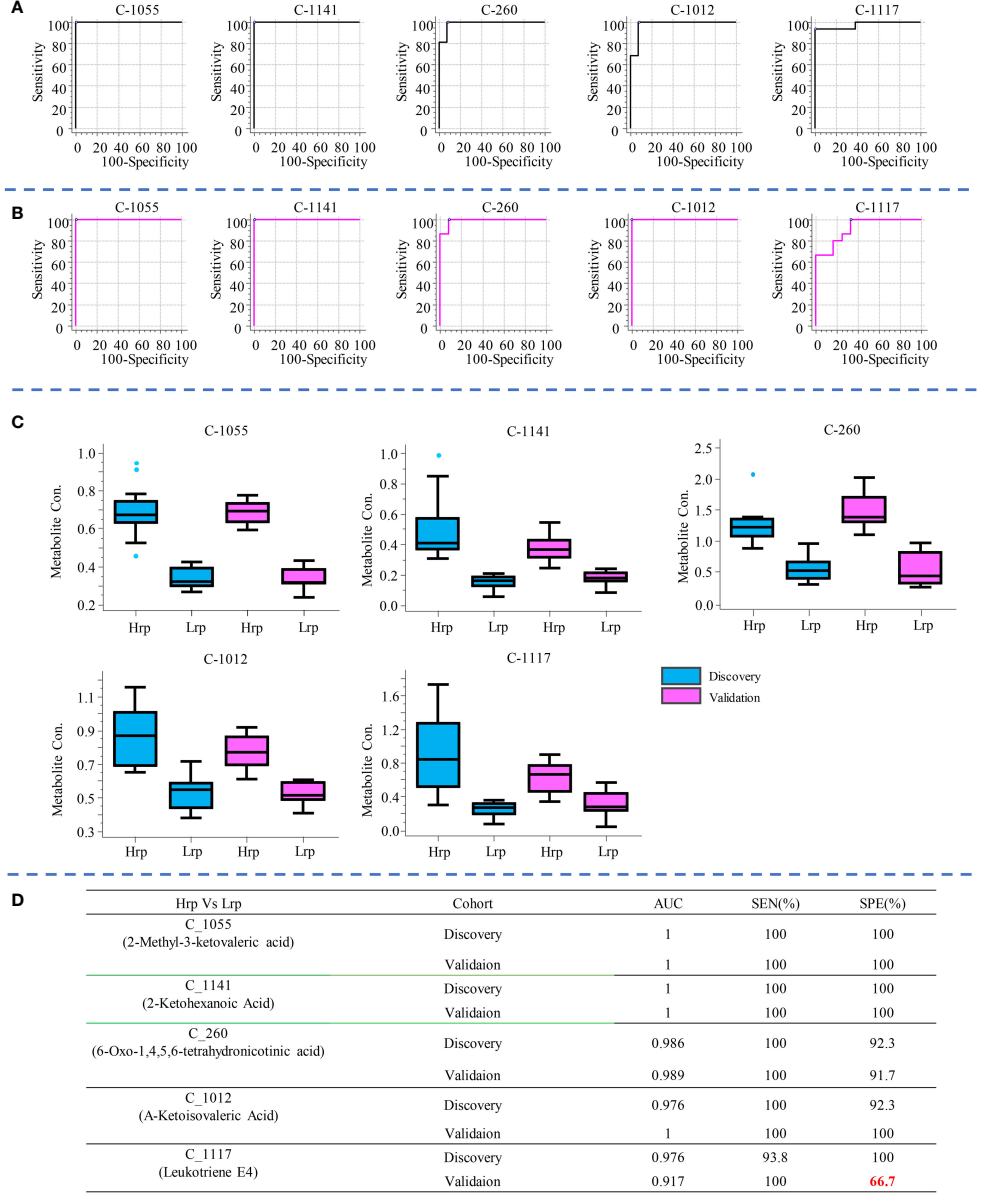
The five metabolites with AUC values greater than or equal to 0.9, demonstrating high sensitivity and specificity, are shown for the discovery cohort **(A)** and the validation cohort **(B)**. Box plot diagrams for each of these five metabolites are presented separately for the discovery and validation cohorts in **(C)**. The AUC value, sensitivity, and specificity for each metabolite in distinguishing between Hrp and Lrp groups are provided in **(D)**.

In clinical practice, there is still no effective clinical predictive indicator for predicting the antiviral treatment efficacy in chronic hepatitis B patients. It is indeed desirable to identify indicators that can predict the efficacy of antiviral treatment in patients before the therapy begins. By comparing the differences between the two groups at T0 (baseline), it may be possible to find potential predictive biomarkers. As shown in the volcano plots generated for this comparison (**Figures 3A, B**), a total of 227 and 162 significant metabolites were altered in the discovery and validation cohort, respectively. We focused on the tier 1 and tier 2 metabolites, as shown in **Supplementary Table S2**. The most abundant metabolite families found to be altered in both cohorts were carboxylic acids and derivatives, keto acids and derivatives, and fatty acids, as shown in **Figures 3C, D**. We concentrated on metabolites predictive of treatment efficacy. **Figure 4** shows the five metabolites with AUC values of greater than or equal to 0.9, with high sensitivity and specificity, that exhibited changes in the discovery cohort (**Figure 4A**) and validation cohort (**Figure 4B**). Among them, four metabolites, namely C_1055 (2-methyl-3-ketovaleric acid), C_1141 (2-ketohexanoic Acid), C_260 (6-oxo-1,4,5,6-tetrahydronicotinic acid), and C_1012 (α-ketoisovaleric acid), demonstrated exceptionally high sensitivity and specificity in both cohorts, nearing 100%. **Figure 4C** shows the boxplots for each of the five metabolites, separately for the discovery and validation cohorts. However, C_1117 (leukotriene E4) stands out as an anomaly. Its specificity in the validation cohort dropped from 100% to 66.7%, although the AUC value for leukotriene E4 remained significantly high, surpassing 0.9 (**Figure 4D**).

**Figure 5:**
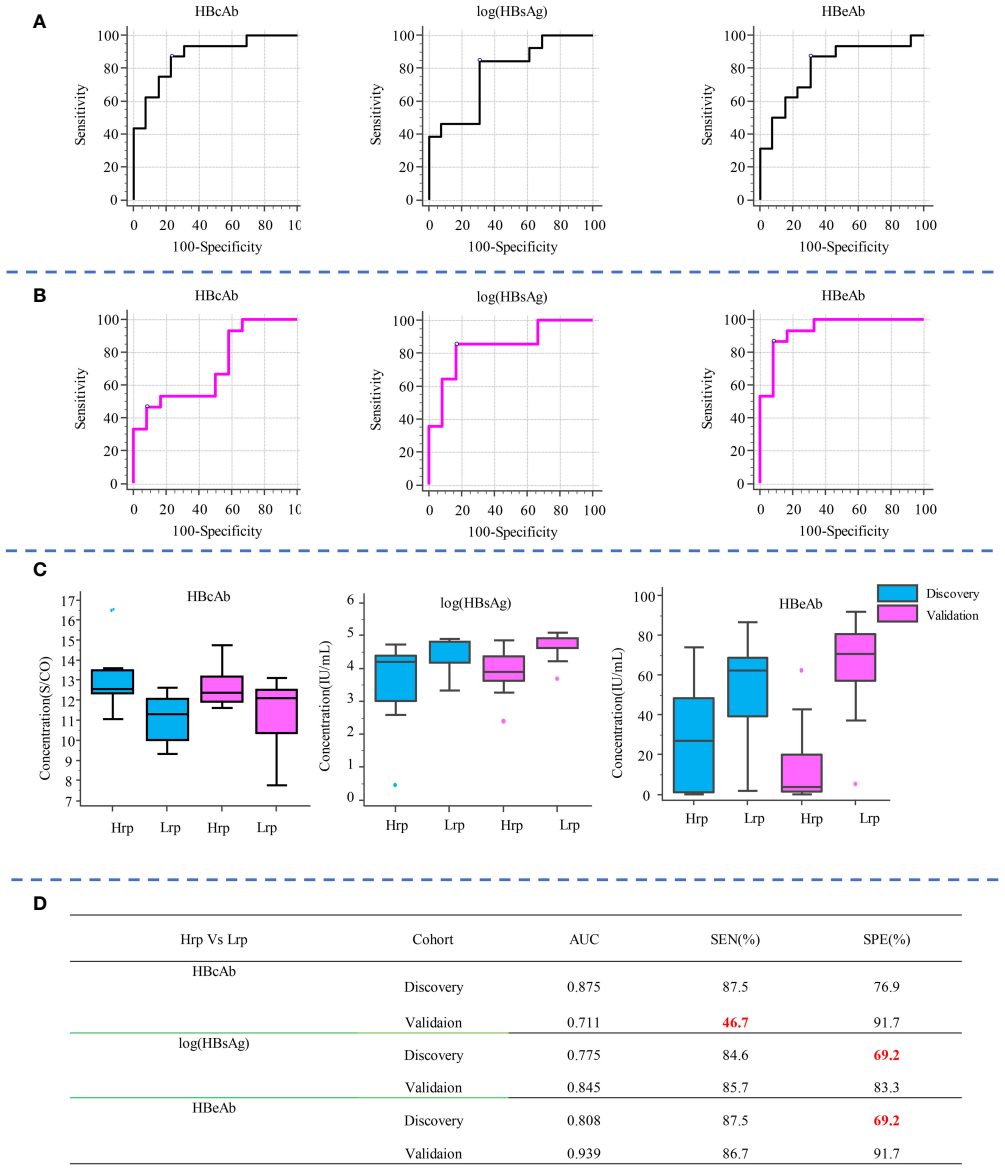
Diagnostic potential of the top three clinical indicators, shown by AUC values and sensitivity and specificity in predicting treatment outcomes in the discovery **(A)** and validation **(B)** cohorts. Box plot diagrams for these indicators across both cohorts are shown in **(C)**. The AUC, sensitivity, and specificity values for each clinical indicator in distinguishing between Hrp and Lrp groups are detailed in **(D)**.

Similarly, to compare the predictive ability of metabolites with clinical indicators, we selected the top three clinical indicators based on their AUC values and evaluated their sensitivity and specificity in predicting treatment outcomes, as shown in **Figure 5**. Compared with clinical predictive indicators, the metabolites exhibit a more robust predictive ability compared to clinical indicators. For instance, the sensitivity of HBcAb decreased from 87.5% in the discovery cohort to 46.7% in the validation cohort. The specificity of HBsAg increased from 69.2% in the discovery cohort to 83.3% in the validation cohort, while the specificity of HBeAb increased from 69.2% in the discovery set to 91.7% in the validation set, indicating the inconsistency and unreliability of clinical indicators in predicting treatment outcomes.

The above comparison of the performance of metabolite predictors and clinical indicators indicates that metabolic biomarkers possess not only high sensitivity and specificity in predicting therapeutic efficacy, but also exhibit superior robustness, stability, and reproducibility, compared to the use of clinical indicators as predictive biomarkers.

#### 3.4.2 Longitudinal study: analyzing the dynamic changes in the metabolome during antiviral treatment

**Figure 6:**
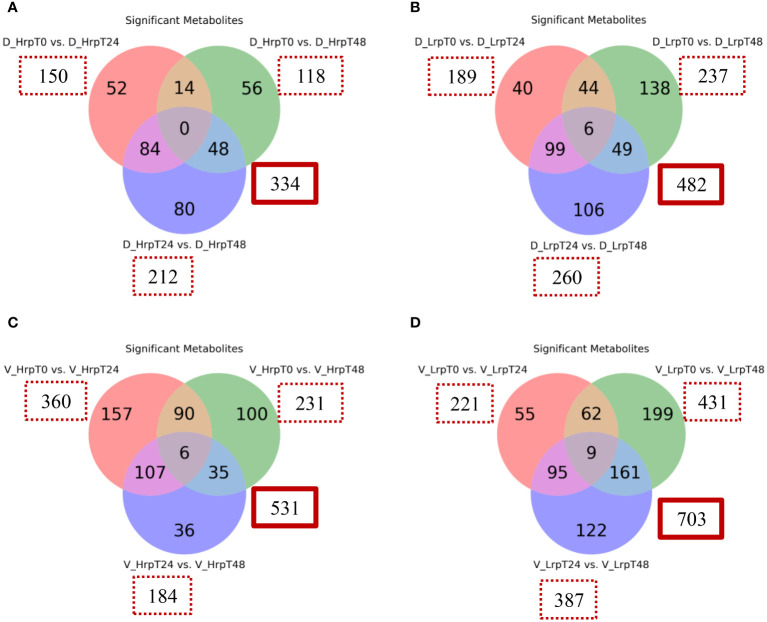
Venn diagrams illustrate the count of significantly altered metabolites from binary comparisons at treatment time points (T0 vs T24, T0 vs T48, T24 vs T48) for high (Hrp) and low (Lrp) immune response groups: **(A)** for Hrp and **(B)** for Lrp in the discovery cohort, followed by **(C)** for Hrp and **(D)** for Lrp in the validation cohort. Each circle in the Venn diagram represents a binary comparison between two time points (T0 vs. T24, T0 vs. T48, and T24 vs. T48), and the overlapping regions indicate the number of metabolites that are significantly changed in multiple comparisons. The analysis applies a fold change threshold of ≥ 1.5 or ≤ 0.67 and controls the false discovery rate (FDR) at 5% with a q-value ≤ 0.05.

We have examined the temporal changes in metabolic profiles within a cohort undergoing antiviral treatment, tracking the evolution of these profiles from the initial baseline to the end of the treatment period. **Figure 6** presents a Venn diagram illustrating the metabolites within each group that exhibited significant changes at different time points (T0 vs T24, T0 vs T48, T24 vs T48) for two groups in both cohorts. The analysis applied a fold-change threshold of either ≥ 1.5 or ≤ 0.67, with a false discovery rate set at 5% (q-value of ≤ 0.05).

In the discovery cohort, intra-group analysis revealed that the Hrp group exhibited 334 differential metabolites throughout the course of treatment, in contrast to the 482 metabolites identified in the Lrp group. In the validation cohort, intra-group analysis showed that the Hrp group exhibited 531 differential metabolites throughout the course of treatment, in contrast to the 703 metabolites identified in the Lrp group. Notably, a more pronounced intra-group alteration in the metabolite profile was observed in the Lrp group in both cohorts, signifying a significant shift in the plasma metabolic pattern of patients with chronic hepatitis B (CHB) within this group during the stages of antiviral therapy.

**Figure 7:**
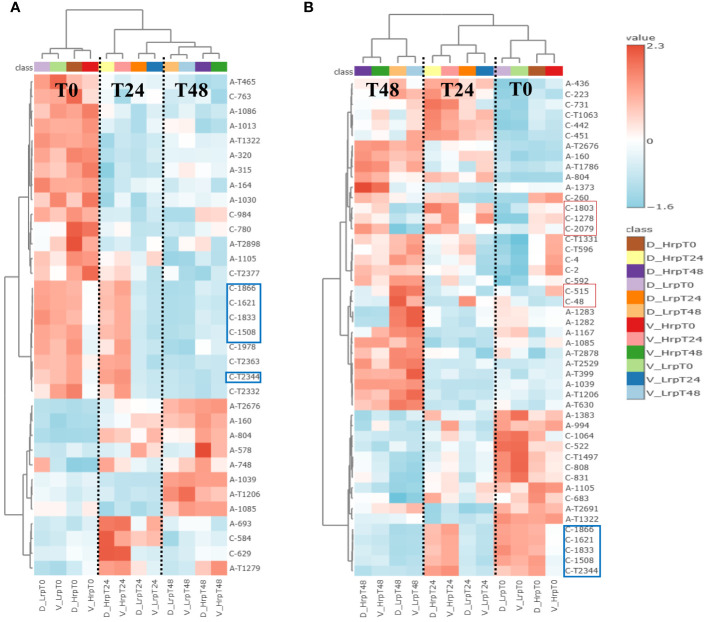
**(A)** Heatmap displays metabolites with significant changes consistent across both discovery and validation cohorts in the high immune response (Hrp) group during antiviral treatment. **(B)** Heatmap shows metabolites consistently altered in both cohorts for the low immune response (Lrp) group throughout the treatment period.

We also focused on the metabolites identified within each group over time, as detailed in **Supplementary Table S3**. We compared metabolites that changed significantly during the treatment in both cohorts to distinguish shared and unique metabolic alterations. In our analysis of the Hrp group during antiviral treatment, we identified 34 differential common metabolites present in both the discovery and validation sets, with their heatmap displayed in **Figure 7A**. Similarly, in the Lrp group, we found 48 common differential metabolites common to both sets, with their heatmap displayed in **Figure 7B**. Among them, five metabolites, (7S,8S)-DiHODE, octadec-9-ene-1,18-dioic acid, prostaglandin J2 (PGJ2), 9-oxoODE, and 15d-PGJ2, were identified as their common significantly changed metabolites. At week 24 of the treatment, the levels of these metabolites were higher in the Hrp group than in the Lrp group, with a fold change of greater than 2, while their levels continued to decrease in the Lrp group. **Supplementary Figure S2** displays the bar diagrams for these five significant metabolites within both the discovery and validation cohorts at various time points.

## 4 Discussion

The integration of metabolomic analysis into clinical practice holds promise for improving personalized treatment strategies and achieving better clinical outcomes. In this work, we used CIL LC-MS to conduct the metabolomic analysis which provided a comprehensive overview of the metabolic changes in CHB patients during antiviral treatment. The identification of 2,904 peak pairs, with 90.7% being either positively identified or matched, highlights the robustness of this approach in monitoring metabolic alterations. The PCA and PLS-DA analysis of the metabolome data clearly differentiated the metabolic profiles of the Hrp and Lrp groups over time, indicating distinct metabolic trajectories in response to therapy.

The cross-sectional study revealed significant differences in metabolite levels between the Hrp and Lrp groups at baseline, 24 weeks, and 48 weeks. The identification of key metabolites, such as 2-methyl-3-ketovaleric acid and α-ketoisovaleric acid, with high sensitivity and specificity as potential predictive biomarkers is particularly noteworthy (see **Figure 4D**). These metabolites showed superior predictive ability compared to traditional clinical indicators, which exhibited inconsistency and lower reliability (see **Figure 5D**).

The longitudinal analysis provided valuable insights into the dynamic metabolic changes during antiviral therapy. The Hrp group exhibited fewer intra-group metabolic alterations compared to the Lrp group, suggesting a more stable metabolic response to treatment. The identification of common differential metabolites, such as prostaglandin J2 and 15d-PGJ2, which displayed significant changes over time, highlights their potential role in monitoring treatment efficacy. The more pronounced metabolic shifts observed in the Lrp group may indicate a need for alternative or adjunctive therapeutic strategies to enhance treatment effectiveness. The significant changes in metabolites related to fatty acid and carboxylic acid pathways further suggest that these metabolic pathways could be targeted to improve antiviral responses.

Among the significant metabolites, (7S,8S)-DiHODE and 9-oxoODE are lipid molecules derived from linoleic acid, an essential ω-6 fatty acid. They are formed through the action of lipoxygenase enzymes, which catalyze the oxidation of linoleic acid. Both have been demonstrated to possess anti-inflammatory properties and to modulate the immune response (18). Prostaglandin J2 (PGJ2) belongs to the cyclopentenone prostaglandin family, derived from the cyclooxygenase (COX) pathway of arachidonic acid metabolism. PGJ2 and its derivatives are known to have anti-inflammatory properties, capable of inhibiting the expression of inflammatory cytokines and chemokines, thus potentially serving as modulators of inflammatory responses (19). PGJ2 has been observed to regulate cell proliferation and induce apoptosis in various cell types, including cancer cells (20). 15d-PGJ2 is a natural ligand of PPARγ, a nuclear receptor that plays a crucial role in the regulation of lipid metabolism, glucose homeostasis, and inflammation. Through the activation of PPARγ, PGJ2 derivatives can impact a wide array of metabolic and inflammatory processes (21). One study demonstrated that 15d-PGJ2 inhibits neutrophil infiltration during the resolution phase of inflammation, suggesting that 15d-PGJ2 may exert its effects by promoting the death of neutrophils during the resolution process of inflammation (22). Octadec-9-ene-1,18-dioic acid is a long-chain dicarboxylic acid that belongs to the class of fatty acids, specifically to the group of unsaturated dicarboxylic acids. Although some long-chain unsaturated fatty acids and their derivatives have shown beneficial physiological effects on human health, such as anti-inflammatory properties and regulation of the immune system (23), there are no clear research reports in the current public literature regarding the specific physiological functions of octadec-9-ene-1,18-dioic acid within living organisms.

Overall, these metabolites share similar physiological functions related to anti-inflammatory properties, immune modulation, and regulation of metabolic processes. Their increased levels indicate a positive immune response in CHB patients undergoing antiviral treatment. It typically also portends a favorable physiological environment as well as an antiviral effect.

Additionally, the heatmap in **Figure 7B** also displays several other metabolites that are significantly different and quite interesting, as they may be related to immunity and inflammation. **Supplementary Figure S3** shows bar diagrams of the trends of these metabolites such as (C-48, thiocysteine), (C-515, tyrosine), (C-2079, retinoic Acid), (C-1278, alpha-muricholic Acid), and (C-1803, 3beta-hydroxy-delta5-cholenic Acid).

Among them, the levels of thiocysteine and tyrosine were significantly higher in the Lrp group compared to the Hrp group at the end of treatment. Thiocysteine is a sulfur-containing amino acid derivative that is structurally similar to the amino acid cysteine, with the primary difference being the substitution of a sulfur atom for the oxygen atom in the cysteine thiol group, resulting in a thiol-thione group. This modification imparts unique chemical properties to thiocysteine compared to cysteine, especially in terms of its reactivity with metals and other compounds. Mishanina et al. (24) indicated that thiocysteine is a potential precursor of reactive sulfur species (RSS). Under oxidative stress conditions, cysteine may participate in the generation of reactive sulfur species (RSS), such as thiols and sulfur-based free radicals. These reactive sulfur species also play important roles in cellular signaling and regulation (25). Additionally, there tends to be an increase in tyrosine levels as liver fibrosis progresses through its stages (26). This correlation between tyrosine levels and the stage of liver fibrosis has led to the proposal of tyrosine as a potential biomarker for assessing the severity of liver fibrosis. Thus, the elevated levels of tyrosine and thiocysteine in the Lrp group may suggest more advanced stages of fibrosis or inflammation, making the measurement of these metabolites a potentially valuable tool for clinicians in evaluating the progression of liver disease.

The metabolites of the second pattern showed higher levels in the Hrp group compared to the Lrp group throughout the entire treatment process, represented by retinoic acid (C-2079), alpha-muricholic Acid (C-1278), and 3beta-hydroxy-delta5-cholenic Acid (C-1803). Retinoic acid (RA) serves as a metabolic intermediate of vitamin A. RA deficiency is associated with the development of various liver diseases, including nonalcoholic fatty liver disease, chronic hepatitis, liver fibrosis, and liver tumors (27). As the treatment progressed, the RA content in the Hrp group gradually exceeded that in the Lrp group and remained higher until the end of treatment. RA supplementation has shown potential in preventing or treating these conditions by regulating relevant signaling pathways, oxidative stress, and cell differentiation (28). Recent research has demonstrated a substantial correlation between vitamin A levels and the onset and progression of liver injury (29). All-trans retinoic acid treatment reduces bile acid synthesis and potentially hepatic inflammation in PSC patients (30).

Furthermore, the observation that alpha-muricholic Acid and 3beta-Hydroxy-delta5-cholenic acid, both related to bile acid metabolism, are present in higher concentrations in chronic hepatitis B patients with a high level of immune response compared to those with a poor immune response, and that this trend becomes more pronounced over the course of treatment, may reflect a close association between bile acid metabolism and the immune response in patients with chronic hepatitis B. Bile acids are involved in lipid digestion and absorption but also have roles in regulating immune functions. For example, In a reported study (31), the authors demonstrated that bile acids, including cholic acid and chenodeoxycholic acid, can induce the differentiation of monocytes into dendritic cells that produce high levels of interleukin-12 (IL-12), a pro-inflammatory cytokine. This effect was mediated through the activation of the G protein-coupled receptor TGR5, which is expressed on innate immune cells. Other authors (32) also found that increased levels of isoalloLCA in feces were associated with decreased circulating IL-17A, suggesting that bile acid metabolites can influence immune responses by modulating the gut microbiota. Although these studies do not directly mention α-MCA and 3β-HCA, they provided evidence that bile acids and their metabolites can influence immune responses by modulating the gut microbiome and the function of immune cells. These findings suggest that α-MCA and 3β-HCA, as bile acids, may also indirectly regulate the immune system through similar mechanisms.

There are some limitations in this study. The study’s small cohort sizes restrict the statistical robustness of the results. Although potential biomarkers were identified, the study’s retrospective design and absence of prospective validation also hinder the conclusive establishment of these markers as predictive. To address this, future research should include prospective studies in larger cohorts, including samples collected by multiple centers, allowing for enhancing the statistical power and generalizability of our findings and drawing more definitive conclusions. Additionally, longer-term monitoring would enhance understanding of treatment responses. Finally, the mechanistic understanding of how identified metabolites correlate with treatment response remains preliminary, necessitating further investigation into the involved biological pathways.

We recognize that achieving a functional cure, defined by sustained HBsAg loss and undetectable HBV DNA, is the ultimate goal of CHB treatment. We also acknowledge the potential for grouping by functional cure and non-cure followed by serological metabolomics analysis to provide deeper insights into the metabolic profiles associated with different treatment outcomes. However, our retrospective analysis revealed that while most patients could effectively suppress viral DNA and achieve e-antigen seroconversion following antiviral therapy, only a minority attained HBsAg surface antigen conversion, indicating functional cure. Among the 31 enrolled samples in our study (HRP, n=31), only 2 achieved functional cure, while the remaining 29 achieved e-antigen seroconversion. Consequently, our study utilized e-antigen seroconversion and DNA negativity as positive prognostic indicators, highlighting the challenges associated with achieving functional cure and the importance of monitoring these markers for treatment efficacy assessment.

Building upon this experimental foundation, CHB patients with low HBeAg levels are often more likely to achieve clinical cure. We are currently conducting a prospective clinical study where enrolled CHB patients with low HBeAg levels will receive antiviral therapy. We will continue to investigate the plasma metabolomic changes in CHB patients who achieve clinical cure and those who exhibit suboptimal treatment responses. Based on the findings from these studies, we plan to carry out subsequent biological validation experiments.

## 5 Conclusions

In this study, we employed a chemical isotope labeling liquid chromatography-mass spectrometry metabolomics approach to comprehensively profile the amine/phenol and carboxylic acid submetabolomes in patients with chronic hepatitis B. This platform allowed for the quantitative analysis of nearly 3000 metabolites and their changes over the course of antiviral treatment. Several metabolites were identified that could differentiate between patients with high and low response to treatment, demonstrating diagnostic potential. Additionally, a set of metabolites were found to be associated with HBeAg seroconversion. Patients exhibiting favorable treatment responses showed metabolic signatures indicative of activated immune systems and gradually controlled inflammation. These findings provide insights into the molecular mechanisms regulating the hepatic inflammatory response during antiviral treatment. A better understanding of metabolic regulation in this context could help the development of optimized treatment strategies helping more chronic hepatitis B patients achieve functional cures.

While this study was limited to the group treated with ETV and IFN-α, the methodology should be applicable to other treatment groups subjected to different regimens. In future research, we will consider comparing various antiviral treatment regimens (antiviral therapy alone, interferon therapy alone, and combined antiviral and interferon therapy) to assess the specificity of the identified metabolites in predicting treatment outcomes across different therapies.

## Data availability statement

The data presented in the study are deposited in the MetaboLights repository, accession number MTBLS10401.

## Ethics statement

The studies involving humans were approved by Human Ethics Committee of the First Affiliated Hospital of Zhejiang University, Hangzhou, China. The studies were conducted in accordance with the local legislation and institutional requirements. The participants provided their written informed consent to participate in this study.

## Author contributions

DC: Conceptualization, Methodology, Writing – original draft, Writing – review & editing, Data curation, Formal analysis, Investigation, Validation. YL: Data curation, Formal analysis, Investigation, Writing – review & editing. JL: Data curation, Formal analysis, Investigation, Writing – review & editing. JY: Data curation, Formal analysis, Investigation, Writing – review & editing. LL: Writing – review & editing, Conceptualization, Funding acquisition, Methodology, Supervision, Writing – original draft. LJL: Writing – review & editing, Conceptualization, Funding acquisition, Supervision.
