# Supplementary material for: Plasma metabolome analysis for predicting antiviral treatment efficacy in chronic hepatitis B: diagnostic biomarkers and therapeutic insights
Source: Front Immunol. 2024 Jul 12;15:1414476. doi: 10.3389/fimmu.2024.1414476 (PMC11272971; doi:10.3389/fimmu.2024.1414476)
Supplement: Supplementary file 1 [file DataSheet_1.pdf]

(A)

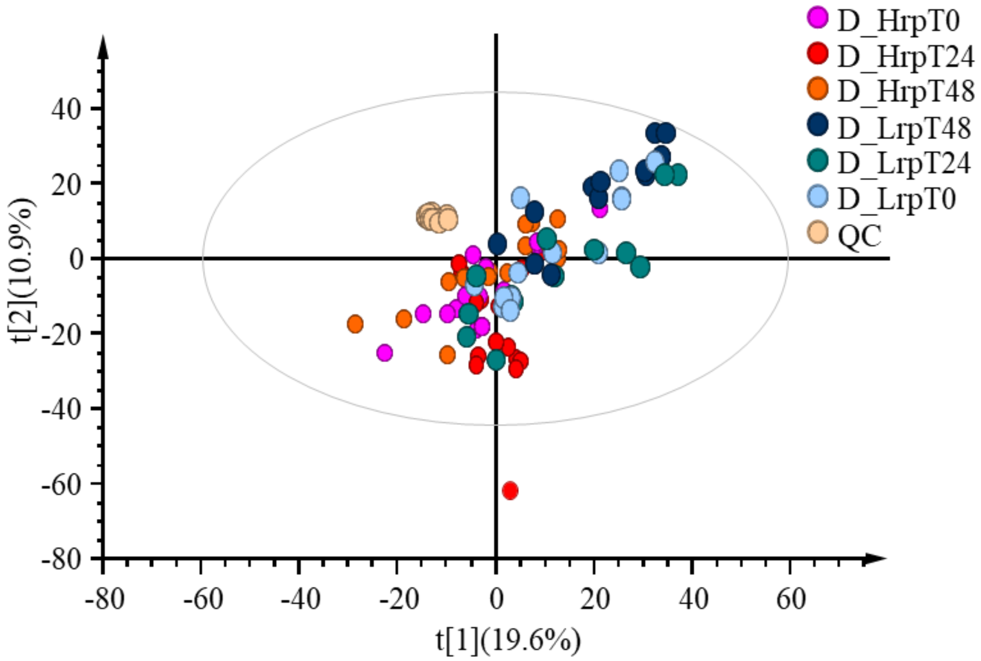

(B)

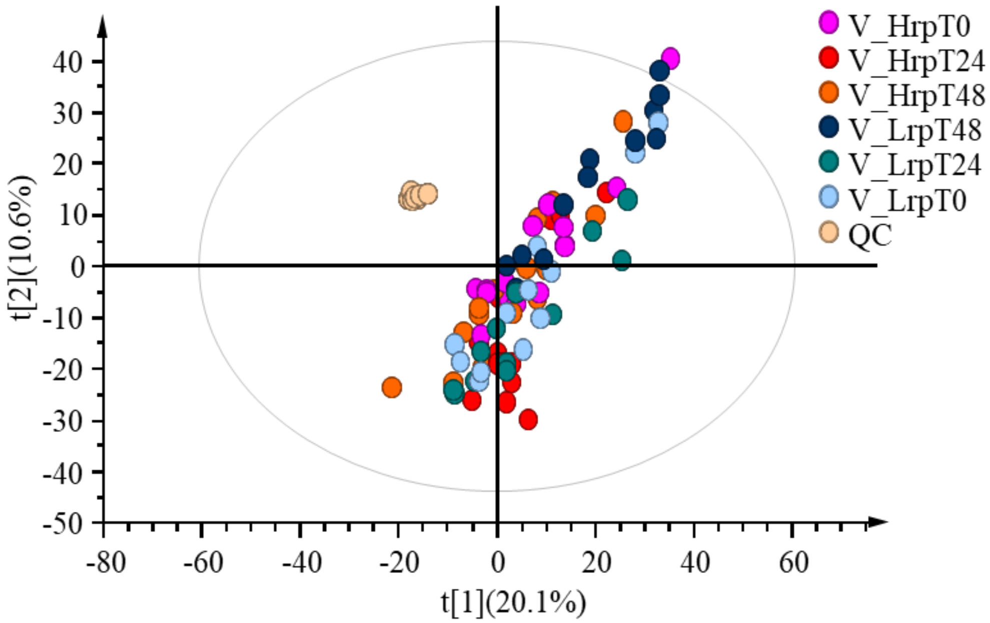

Figure S1. Principal Component Analysis (PCA) scores plot showing the distribution of (A) discovery samples at the time points T0 (baseline), T24 (24 weeks), and T48 (48 weeks), including quality control (QC) samples, and (B) validation samples. (A) R2X=0.645, Q2=0.425 and (B) R2X=0.624, Q2=0.415.

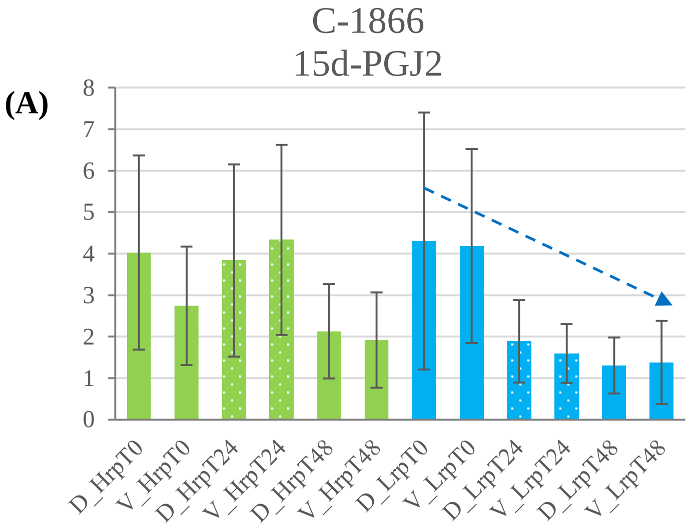

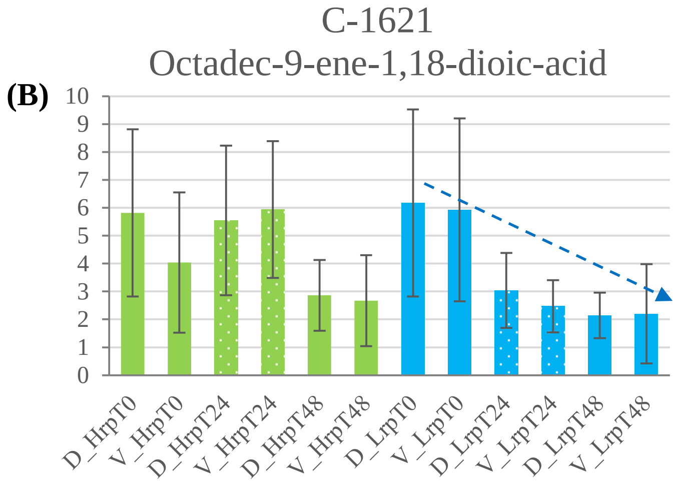

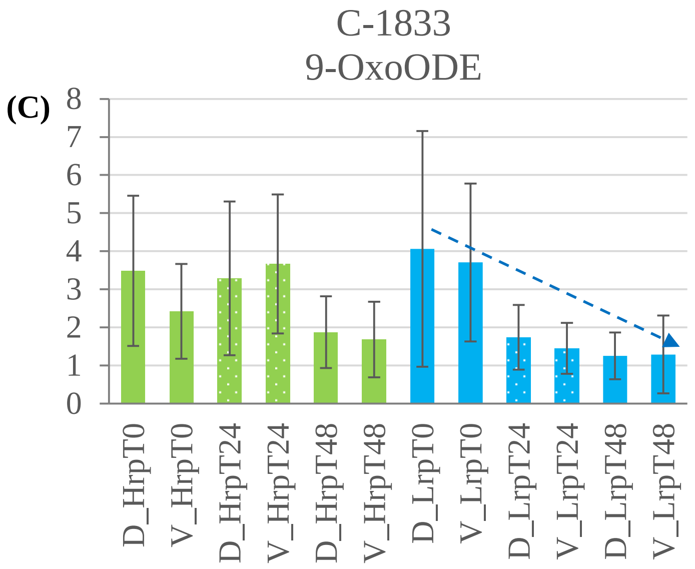

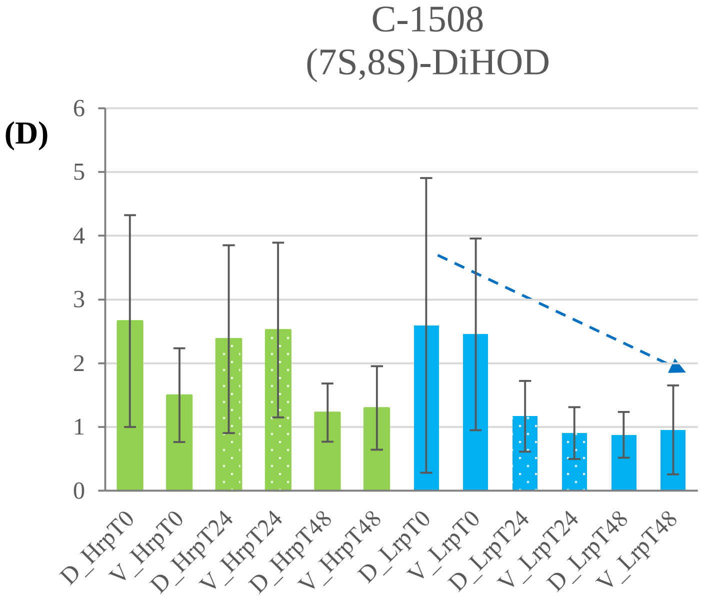

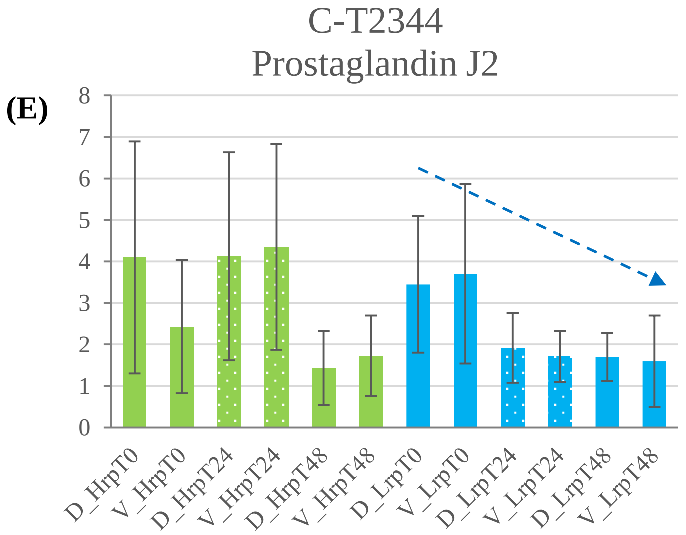

Figure S2. Bar charts of five significant metabolites shared between the Hrp and Lrp groups, observed at different time points in both the discovery and validation cohorts.

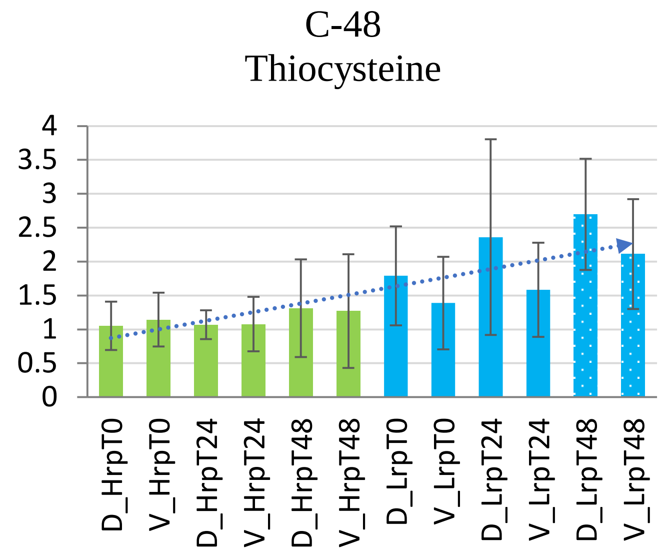

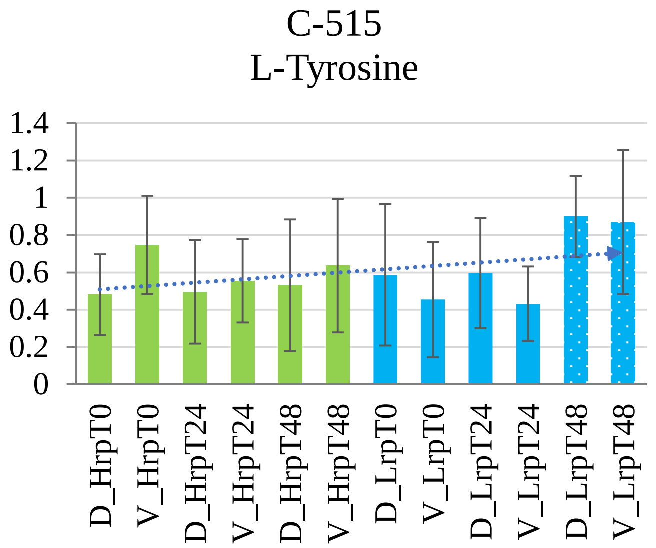

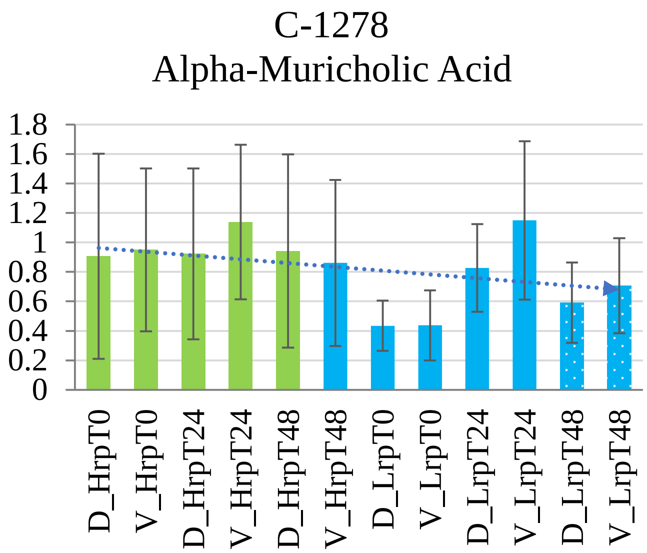

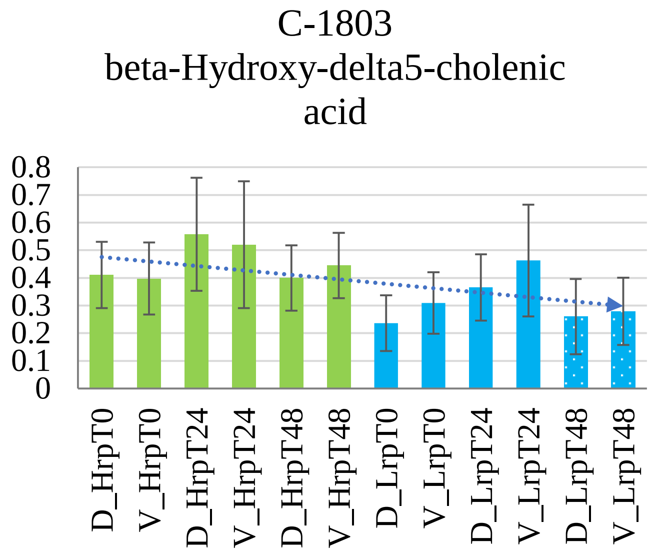

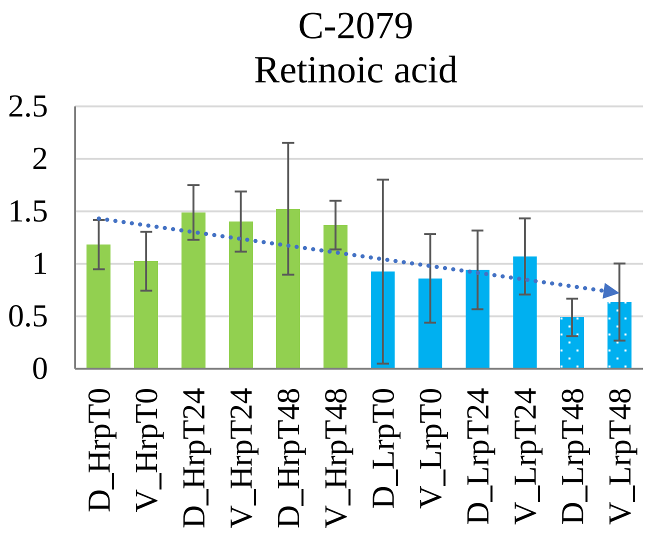

Figure S3. Bar diagrams showing the trends in metabolites levels that are significantly different and potentially related to immunity and inflammation across both discovery and validation cohorts at various time points.
